# Spatial and Spectral Reconstruction of Breast Lumpectomy Hyperspectral Images

**DOI:** 10.3390/s24051567

**Published:** 2024-02-28

**Authors:** Lynn-Jade S. Jong, Jelmer G. C. Appelman, Henricus J. C. M. Sterenborg, Theo J. M. Ruers, Behdad Dashtbozorg

**Affiliations:** 1 Image-Guided Surgery, Department of Surgery, Netherlands Cancer Institute, Plesmanlaan 121, 1066 CX Amsterdam, The Netherlands; 2 Department of Nanobiophysics, Faculty of Science and Technology, University of Twente, Drienerlolaan 5, 7522 NB Enschede, The Netherlands; 3 Faculty of Science, Vrije Universiteit Amsterdam, De Boelelaan 1111, 1081 HV Amsterdam, The Netherlands

**Keywords:** breast-conserving surgery, hyperspectral imaging, resection margin assessment, breast tissue, deep learning, super-resolution reconstruction

## Abstract

(1) Background: Hyperspectral imaging has emerged as a promising margin assessment technique for breast-conserving surgery. However, to be implicated intraoperatively, it should be both fast and capable of yielding high-quality images to provide accurate guidance and decision-making throughout the surgery. As there exists a trade-off between image quality and data acquisition time, higher resolution images come at the cost of longer acquisition times and vice versa. (2) Methods: Therefore, in this study, we introduce a deep learning spatial–spectral reconstruction framework to obtain a high-resolution hyperspectral image from a low-resolution hyperspectral image combined with a high-resolution RGB image as input. (3) Results: Using the framework, we demonstrate the ability to perform a fast data acquisition during surgery while maintaining a high image quality, even in complex scenarios where challenges arise, such as blur due to motion artifacts, dead pixels on the camera sensor, noise from the sensor’s reduced sensitivity at spectral extremities, and specular reflections caused by smooth surface areas of the tissue. (4) Conclusion: This gives the opportunity to facilitate an accurate margin assessment through intraoperative hyperspectral imaging.

## 1. Introduction

Breast-conserving surgery (BCS) is considered the preferred treatment for women suffering from early-stage breast cancer. With this type of surgery, surgeons aim for a complete tumor resection while achieving the highest cosmetic result. However, in most cases, the tumor is not visible or palpable, which imposes hard challenges for the surgeons who heavily rely on visual and tactile feedback when it comes to the discrimination of tumor and healthy tissue. Consequently, previous studies have reported incomplete tumor resections usually in the range from 5% to 20% of the surgeries depending on national guidelines [1,2,3,4,5,6]. The presence of tumor residuals necessitates an adjuvant treatment, leading to a significant deterioration of the patient’s quality of life. Furthermore, it is associated with an elevated risk of recurrent breast cancer [7,8]. As a result, the need for innovative treatment possibilities has risen and has led to a diversity of novel techniques, e.g., diffuse reflectance spectroscopy, microcomputed tomography, optical coherence tomography, and photoacoustic imaging, with the purpose of supporting surgeons in distinguishing between various tissue types during BCS [9,10,11]. Despite the several techniques, none of them have yet been implemented in the surgical workflow.

Hyperspectral imaging (HSI) is a promising optical imaging technique, which can, due to its properties of being non-invasive, radiation-free, and not requiring any contrast agents, be implicated as a real-time feedback system during surgery to improve the outcome of BCS. With this technique, a tissue sample undergoes illumination from a broadband light source. Subsequently, due to variations in the molecular composition of cells within the tissue, multiple absorption and scattering events occur with the incident light. The diffuse light that is reflected is then captured and processed by a camera sensor that measures the different light intensities, resulting in a spectrum with various wavelengths for each pixel. The shape of this acquired spectrum is indicative of the tissue’s composition and morphology, enabling the discrimination between different tissue types. The resulting image of the tissue, with an associated spectrum for each pixel, is called a three-dimensional hypercube, containing both spatial and spectral information.

These images can be analyzed using machine and deep learning methods to assess the margin (surgical surface) of the resected tissue as either healthy or tumorous. Deep learning methods are well suited for hyperspectral image analysis tasks due to their ability to effectively handle complex and high-dimensional input data [12,13,14,15,16]. Moreover, they are capable of automatically extracting essential features, eliminating the need for specialized expertise when dealing with the data. Hence, the characteristics of HSI combined with deep learning analysis methods has major advantages compared to conventional margin assessment techniques, i.e., frozen section analysis, imprint cytology, and specimen radiography [12,17].

The application of HSI with deep learning has been employed for various medical diagnostic purposes, e.g., blood vessel and nerve detection [18], tissue perfusion assessment [19], and wound evaluation [20]. Furthermore, it has also frequently been used for cancer detection. Studies that focus on the head and neck, colon, as well as breast, show promising results, with an accuracy of up to 81% for head and neck cancer [21,22], 88% for colon cancer [21], and 98% for breast cancer [12,13].

Although HSI shows a high amount of promise, it still leaves room for improvement. Particularly, when taking the step toward clinical translation. To date, in all prior studies utilizing HSI for breast cancer detection, the measurements were conducted with a line-scanning hyperspectral camera on ex vivo resection specimens, known as lumpectomy specimens, following surgery. However, our ultimate goal is to employ real-time HSI on in vivo tissue directly within the surgical workflow in the operating room.

To make HSI usable during surgery, it should be fast and capable of yielding high-quality images. However, there exists a trade-off between image quality and data acquisition time, as higher resolutions result in longer acquisition times. Additionally, extended acquisition times elevate the risk of motion artifacts, while it is possible to obtain relatively high-resolution images using a line-scanning hyperspectral camera, this will come at the expense of prolonged acquisition times (more lines should be scanned) and the feasibility of using such bulky cameras for intraoperative surgery. A snapshot hyperspectral camera, on the other hand, could be a good alternative as it provides a faster (the entire scene can be captured in one shot) and more compact solution. Nonetheless, it is important to note that these cameras typically acquire lower-resolution images with fewer wavelength bands compared to line-scanning cameras [23,24].

Super-resolution reconstruction methods are focused on enhancing the resolution and quality of an image using various computational techniques. The process involves taking a lower-resolution image as input and producing a higher resolution version of it. Several approaches have been developed, such as Bayesian estimation [25,26,27], matrix factorization [28,29], and deep learning based methods [30,31,32,33]. Although the Bayesian estimation and matrix factorization are showing great improvement in reconstruction, the more recent approach of deep learning methods is outperforming the previous methods. In particular, Zhang et al. [33] used a neural network, called the SSR-NET, in geoscience to reconstruct satellite hyperspectral images. In their study, the authors demonstrated that by using a multispectral image as the high-resolution image, and fusing it with a low-resolution hyperspectral image, the neural network offers the ability to reconstruct a high-resolution hyperspectral image.

This method of reconstructing a high-resolution hyperspectral image by means of a neural network offers the advantage of a reduced processing time, making it particularly advantageous for surgical procedures; however, it has thus far been employed with datasets outside the medical field. When applying this method to our application, it becomes imperative to consider the clinical conditions under which hyperspectral image reconstruction will occur. In our scenario, it is essential to conduct the reconstruction using hyperspectral images captured by a snapshot camera, as this type of camera can be more easily integrated into the surgical workflow. Additionally, when working with hyperspectral images of breast lumpectomy specimens in the context of breast-conserving surgery, we must anticipate potential blurring caused by motion artifacts. This includes the patient’s respiration, surgical field manipulation, and rapid camera movements, all of which can impact the quality of the image.

On top of that, it is important to consider other common challenges encountered in the application of HSI. These challenges include issues such as dead pixels on the camera sensor, noise caused by the sensor’s reduced sensitivity at spectral extremities, and specular reflections due to smooth surface areas of the tissue. As these issues affect the hyperspectral image quality and could have negative implications for the analysis process, the adoption of the SSR-NET network emerged as a promising solution to overcome them by reconstructing high-resolution hyperspectral images.

Thus, in this study, we present a novel reconstruction framework to obtain high-resolution hyperspectral images during BCS. Our framework employs super-resolution reconstruction to improve the quality of low-resolution hyperspectral images by incorporating RGB images from a regular color camera, and taking advantage of their high-resolution. As a regular color camera is relatively cheap, and can be easily synchronized with a snapshot camera, this enables a fast data acquisition with HSI during surgical procedures without compromising image quality. On top of that, this advancement provides the opportunity to ultimately facilitate an accurate margin assessment through intraoperative hyperspectral imaging. Because a comprehensive breast lumpectomy dataset obtained through a snapshot camera is unavailable, our approach uses an existing breast lumpectomy dataset collected with a high-resolution line-scanning camera. With these data, we can establish a process for generating both low-resolution snapshot images and high-resolution RGB images. With these synthetically made images as input, we demonstrate the capability of our framework to reconstruct high-resolution hyperspectral images of breast lumpectomy specimens while effectively handling blur, noise, dead pixels, and specular reflections.

## 2. Methodology

In this section, we present our framework for reconstructing high-resolution hyperspectral images of breast lumpectomy specimens using hyperspectral images from a low-resolution snapshot camera and RGB images from a regular high-resolution color camera as input. Due to the lack of a snapshot lumpectomy dataset, we introduce an alternative approach, which explains how we synthetically generated low-resolution snapshot and high-resolution RGB images from an existing ex vivo lumpectomy dataset. First, we will describe this dataset in more detail. Thereafter, we will delve into our methodology, which elaborates on the generation of the synthetic images and explains how we trained the reconstruction network. Finally, we will provide an overview of the various experiments conducted to evaluate the effectiveness of the reconstruction network on an independent test set.

### 2.1. Breast Lumpectomy Dataset

In this study, a hyperspectral dataset is used that was acquired on lumpectomy specimens from female patients who underwent primary breast-conserving surgery at the Netherlands Cancer Institute—Antoni van Leeuwenhoek hospital. The data acquisition was performed immediately after surgery. The experiments were approved by the Institutional Review Board of The Netherlands Cancer Institute—Antoni van Leeuwenhoek hospital, and were conducted in accordance with the Declaration of Helsinki. Based on the Dutch Medical Research Involving Human Subjects Act (WMO), it was not required to obtain written informed consent from the patients.

The dataset consisted of 50 hyperspectral images captured from the lumpectomy specimens of different patients, all taken from one side. The images were acquired using a line-scanning hyperspectral camera (Specim, Spectral Imaging Ltd., Oulu, Finland, PFD-CL-65-V10E, linear CMOS sensor), including a spectral range between ∼400–1000 nm (384 bands), a spectral resolution of 3 nm, and spatial resolution of 0.16 mm per pixel. The images were calibrated as described in [15].

### 2.2. Framework for Spatial–Spectral  Reconstruction

We introduce our spatial–spectral reconstruction framework to reconstruct high-resolution hyperspectral images of breast lumpectomy specimens from low-resolution hyperspectral images and high-resolution RGB images. Besides this, our method also accounts for blurred hyperspectral images as a result of motion artifacts during surgical procedures and other issues commonly encountered in the application of HSI, such as dead pixels, noise, and specular reflections.

#### 2.2.1. Generating Snapshot and RGB Images

The hyperspectral image is represented as a three-dimensional data structure, often referred to as a hypercube. Here, the spatial information is stored in the first two dimensions, denoted as *X* and *Y*, while the spectral information is encapsulated within the third dimension, referred to as *Z*. Given that the acquired line-scan hyperspectral images from the breast lumpectomy dataset possess high spatial–spectral resolution (HR-HSI), we use them to generate synthetic low-resolution hyperspectral images and high-resolution RGB images. Furthermore, they also serve as reference images (ground-truth) for training and testing the proposed pipeline. By using the line-scan hyperspectral images, we intentionally generated low spatial but high spectral resolution hyperspectral images (LR-HSI), and high spatial but low spectral resolution RGB images (HR-RGB) as inputs for the framework. The synthetically made LR-HSI represent images acquired from a low-resolution hyperspectral camera, for example snapshot camera, and the HR-RGB represent images acquired with a regular high-resolution color camera. To generate the LR-HSI, we applied bilinear downsampling to the reference image. For the HR-RGB, we extracted three spectral bands at specific wavelengths, equivalent to the RGB channels, from the reference image. Section 2.2.3 provides the details of this approach.

#### 2.2.2. Reconstruction Network

**Figure 1:**
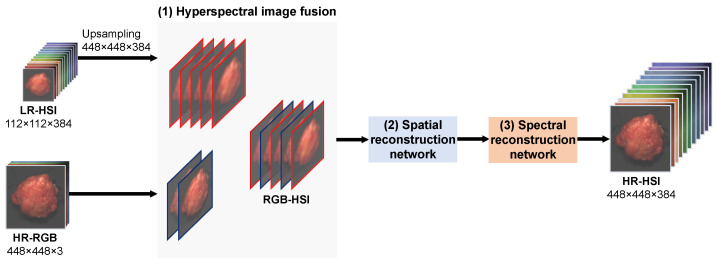
Pipeline of the reconstruction network. The reconstruction network comprises three stages. In the first stage, it takes as input a low spatial but high spectral resolution hyperspectral image (LR-HSI) from, for instance, a snapshot camera and a high spatial but low spectral resolution RGB image (HR-RGB) from a regular color camera. These two inputs are subsequently fused to create a preliminary RGB-HSI image. To achieve this, the LR-HSI is upsampled to a dimension of 448 × 448. Following the fusion process, the RGB-HSI is then processed through the second and third stages. In these stages, the network is trained to independently reconstruct the spatial and spectral information of the image using the reference image as ground-truth. This ultimately produces a hyperspectral image with a high spatial and spectral resolution (HR-HSI).

The architecture of the reconstruction network, based on the paper of Zhang et al. [33], is depicted in Figure 1 and can be described as a straightforward convolutional neural network (CNN). It consists of the following three different stages: (1) hyperspectral image fusion; (2) spatial reconstruction; and (3) spectral reconstruction. Each of these stages is briefly explained and the details of this network are given in Table 1:

1. Hyperspectral Image Fusion

The LR-HSI was upsampled using bilinear interpolation to match the spatial dimensions of the HR-RGB. Following this, we concatenated the LR-HSI and HR-RGB to create a preliminary RGB-HSI image. Within this image, the spectral bands from the LR-HSI are replaced with their corresponding spectral bands from the HR-RGB, preserving the most relevant spectral and spatial information from both the LR-HSI and HR-RGB, respectively. After the fusion process, a convolutional layer was employed to transmit the information of the initial RGB-HSI to the following stages of spatial and spectral reconstruction, followed by the application of a non-linear activation function, namely, the rectified linear unit (ReLU).

2. Spatial Reconstruction

The purpose of this stage is to learn the network to reconstruct the spatial information using the spatial features from the preliminary RGB-HSI. To achieve this, an additional convolutional layer was implemented in the CNN, functioning as the spatial reconstruction network. To optimize the reconstruction between the RGB-HSI and reference HR-HSI, a mean squared error (MSE) loss function was incorporated. This loss function primarily concentrated on spatial intensity variations between neighboring pixels along both the X and Y direction for each wavelength band in the RGB-HSI and reference HR-HSI.

3. Spectral Reconstruction

Following the spatial reconstruction process, the preliminary reconstructed HR-HSI was passed to the last stage. In this stage, a convolutional layer was also incorporated but this one functioned as the spectral reconstruction network to extract the most relevant spectral features from the preliminary reconstructed HR-HSI. An MSE loss function was used to consider the spectral intensity differences between adjacent wavelength bands among pixels in the preliminary reconstructed HR-HSI and reference HR-HSI. After spectral reconstruction, the final reconstructed HR-HSI was optimized once more using a fusion loss function [33]. The overall loss comprised the sum of the spatial, spectral as well as the fused loss.

**Table 1:** Details of the reconstruction network demonstrated in Figure 1. In each stage, a convolutional layer with a kernel size of 3 × 3 is employed with a height and width stride of 1. *l* and *L* represent the number of input and output channels, respectively.

Input	Architecture	Loss Function
RGB-HSI	Conv(3 × 3, *l*, *L*) Conv(3 × 3, *l*, *L*) Conv(3 × 3, *l*, *L*)	- MSE (spatial) MSE (spectral) + fusion

#### 2.2.3. Implementation

The dataset was partitioned into a train (60%), validation (20%), and test set (20%), while ensuring that all images from each patient remained grouped together within their respective sets. We validated the proposed method using both the peak signal-to-noise ratio (PSNR) and spectral angle mapper (SAM) as evaluation metrics (Section 2.4). The images in the training, validation, and test sets were cropped in the center to a size of 448×448. The center of the image was taken to maintain the entire size of the lumpectomy specimen while excluding unnecessary background pixels. We generated the LR-HSI by using a Gaussian filter of kernel size 5×5 and a standard deviation (std) of 10. The downsampling factor was set to 4, yielding images with the size of 112×112 with 384 wavelength bands. For the HR-RGB images, we selected the bands at a wavelength of 650, 532, and 473 nm, congruent to the bands of the RGB-range. The model was trained during 10,000 epochs with the Adam optimizer and a learning rate of 0.0001. The network was implemented using PyCharm 2023.1.2, Python 3.11, and Pytorch 1.3.0 library on a computer workstation with an NVIDIA GeForce GTX 1080 Ti.

### 2.3. Experiments

Once the reconstruction network was trained, we conducted a series of experiments on the test set. These experiments were designed to assess the model’s performance under various issues commonly encountered during the analysis of hyperspectral images. Considering the adverse effects of these issues on the hyperspectral image quality and their impact on the analysis process, we addressed them using the proposed framework. The different experiments are listed as follows:

#### 2.3.1. Blurring

During the surgery, various movements may occur as hyperspectral images are being captured, including the patient’s respiration, manipulation of the surgical field, and rapid camera movements. Given that hyperspectral cameras are built with a fixed focus for a particular distance or depth range, these occurrences may introduce motion artifacts and cause the hyperspectral images to become blurred or out of focus. To replicate these scenarios, we artificially applied a Gaussian blur to the hyperspectral images. Gaussian filters with different kernel sizes of 5×5, 15×15, 25×25, and 35×35, were applied (all with a standard deviation of 10) to simulate the different levels of motion artifacts.

#### 2.3.2. Noise

Depending on the design and technology, hyperspectral camera sensors may have a lower sensitivity at specific wavelength ranges, resulting in more noise at these wavelength bands, which reduces the overall quality of the image [34]. Although we also encounter this problem with our hyperspectral camera, particularly within the shorter wavelengths of our spectral range (400–450 nm), we synthetically created different levels of noise at the longer wavelengths (980–1000 nm) to illustrate the effect on the hyperspectral images and associated spectra. We introduced uniformly distributed random noise at different variances of 0.01, 0.03, 0.05, and 0.07.

#### 2.3.3. Dead Pixels

Hyperspectral cameras may experience a 1% chance of encountering a dead pixel within their sensors, leading to the unfortunate loss of important data [35]. Usually, these defective pixels are located in arbitrary spatial positions. Additionally, they often produce black pixels and erroneous intensity peaks referred to as spikes. To address this issue, we introduced black pixels with a value of 0 along the entire spectral range in the hyperspectral images of the test set. Subsequently, we utilized the reconstruction network to restore these pixels.

#### 2.3.4. Specular Reflection

Light captured by the sensor after bouncing off the sample’s surface is commonly referred to as specular reflection, resembling a mirror-like effect when the surface is smooth. Usually, this type of reflection manifests as a few saturated pixels within the image. When the pixels are saturated, the captured signal is affected, resulting in a loss of valuable information within these pixels. Specular reflection is often also observed in tissue samples. In a surgical setting, this could distort the analysis of hyperspectral imaging. Hence, to explore the potential of the reconstruction network in addressing this concern, we intentionally created four regions characterized by specular reflection, with the respective size of 2×2, 3×3, 4×4, and 5×5 pixels. To simulate spectra of saturated pixels, we constructed a plateau with intensity values exceeding 1 within the wavelength range of 675–705 nm.

### 2.4. Performance Evaluation

We compared the reconstructed HR-HSI images to the upsampled LR-HSI images and the reference HR-HSI images (ground-truth) to evaluate the performance of the reconstruction network on the lumpectomy dataset. To make a fair comparison, we first excluded the black background pixels from the images to avoid any bias of the background on the results. Thereafter, we used the root mean square error (RMSE), peak signal-to-noise ratio (PSNR), relative dimensionless global error synthesis (ERGAS), and spectral angle mapper (SAM) to evaluate the performance of the reconstruction network. The RMSE and PSNR are designed to assess the element-wise reconstruction quality, while the ERGAS and SAM focus on, respectively, the spatial and spectral reconstruction quality.

#### 2.4.1. RMSE

The RMSE is a common metric that can be used to assess the accuracy of the reconstruction network’s performance. It measures the average error between the reference image and the estimated image. The RMSE can be described as follows:

(1) $$RMSE=\sqrt{\frac{\sum_{k=1}^{Z}\sum_{i=1}^{X}\sum_{j=1}^{Y}{\left(G_{k}\left(i,j\right)-R_{k}\left(i,j\right)\right)}^{2}}{XYZ}}$$

where $G_{k}\left(i,j\right)$ and $R_{k}\left(i,j\right)$ are the reflectance values for a pixel (i,j) in wavelength band *k* of, respectively, the reference image (ground-truth image) and the estimated image (i.e., reconstructed HR-HSI or upsampled LR-HSI). A lower RMSE indicates a smaller error and thus a better reconstruction of the image [36,37].

#### 2.4.2. PSNR

Similar to the RMSE, the PSNR is a metric to evaluate the element-wise reconstruction quality of an estimated image with respect to its reference image. It calculates the ratio between the maximum power of the signal in the reference image to the power of the noise that distorts the estimated image per wavelength band [36,38]. The PSNR, expressed in decibels (dB), can mathematically be described as follows:

(2) $$PSNR\left(k\right)=10\log_{10}\left(\frac{max{\left(G_{k}\right)}^{2}}{\frac{1}{XY}{\left(G_{k}-R_{k}\right)}^{2}}\right)$$

After calculating the PSNR values for each wavelength band *k*, the final PSNR can be computed by taking the average over all bands, as shown in Equation (3). A higher PSNR indicates a higher reconstruction quality of the estimated image.

(3) $$PSNR=\frac{\sum_{k=1}^{Z}PSNR\left(k\right)}{Z}$$

#### 2.4.3. ERGAS

The ERGAS calculates, in contrast to the RMSE, a relative error expressed as a percentage [36,39]. The ERGAS is defined as follows:

(4) $$ERGAS=100\frac{XY}{xy}\sqrt{\frac{1}{Z}\sum_{k=1}^{Z}{\left(\frac{RMSE(k)}{\mu (k)}\right)}^{2}}$$

where μ(k) corresponds to the mean value of the reference image for wavelength band *k*. If the ratio $\frac{XY}{xy}$ is equal to one, the reference image and the estimated image have the same spatial resolution. Otherwise, it corresponds to the ratio by which the reference image is downsampled to the LR-HSI image. A smaller ERGAS represents a better performance.

#### 2.4.4. SAM

The SAM measures the spectral similarity between pixels of the estimated and reference image by calculating the angular difference of the corresponding spectra [40]. The SAM, expressed in degrees, is given as follows:

(5) $$SAM=arccos(\frac{\langle G(i,j),R(i,j)\rangle }{∥G(i,j{∥}_{2}∥R\left(i,j\right){∥}_{2}})$$

where the nominator is a multiplication of G(i,j) and R(i,j), which are the spectra of pixel (i,j) in the reference and estimated image, respectively. The denominator represents the inner-product of G(i,j) and R(i,j). The final SAM can be calculated by taking the average over the SAM values of all pixels. The lower the SAM, the higher the spectral similarity.

## 3. Results

In this section, we will provide the outcomes from the reconstruction network applied to the blurring, noise, dead pixels, and specular reflection experiments. It is important to note that for all these experiments, the same reconstruction network was utilized, which had been exclusively trained on blurred low-resolution hyperspectral images and high-resolution RGB images of lumpectomy specimens.

### 3.1. Unblurring

**Figure 2:**
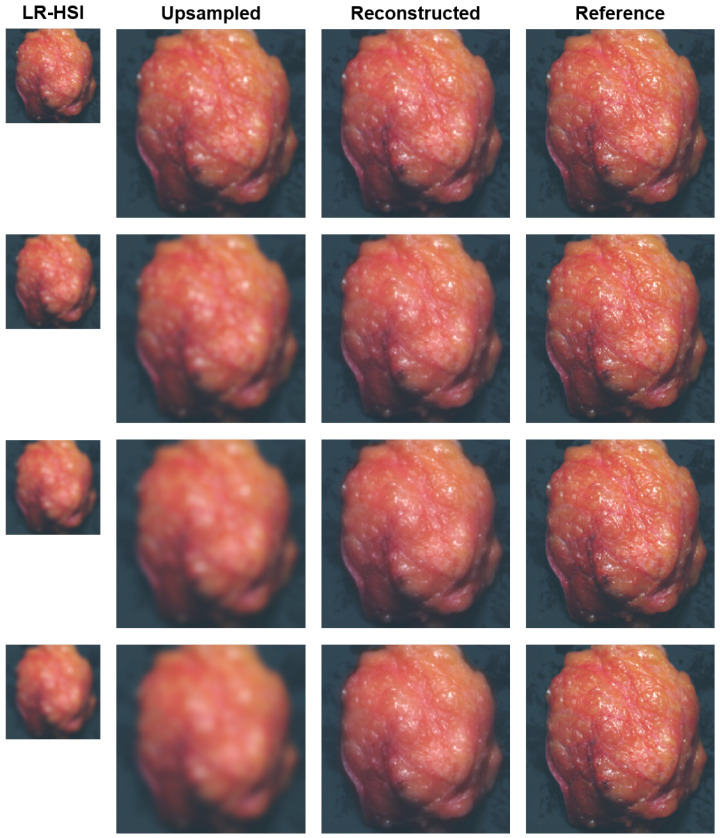
Blurring experiment: example of a lumpectomy specimen after surgery. Pseudocolor images (wavelength bands at 650, 532, 473 nm) are derived from the hyperspectral images. From left to right: Gaussian blurred LR-HSI, upsampled LR-HSI, reconstructed HR-HSI, and reference HR-HSI. The LR-HSI images are blurred with Gaussian filters of different kernel sizes, representing snapshot HSI images in a surgical setting. The upsampled and reconstructed images show the result when, respectively, upsampling and reconstructing the blurred images to high-resolution images. From top to bottom: Gaussian filters with kernel sizes of 5×5, 15×15, 25×25 and 35×35.

Figure 2 shows the results of the blurring experiment for one lumpectomy specimen. A comparison is made between the blurred LR-HSI, upsampled LR-HSI, reconstructed HR-HSI, and their reference HSI images for different Gaussian kernel sizes. The reconstructed images illustrate the model’s capability to transform the blurred low-resolution HSI images of the lumpectomy specimen to high-resolution images, whereas the upsampled images display the outcome of bilinearly upsampling the LR-HSI images only. From the results of Table 2, which gives the average performance metrics of the reconstructed images with respect to the reference images (ground-truth) for all lumpectomy specimens in the test set, it is evident that the performance of the reconstruction decreases as the kernel size of the Gaussian filter increases. Table 2 also gives the average performance metrics of the upsampled images with respect to their reference image. For a kernel size of 5×5, the upsampled images outperform the reconstructed images on all metrics except for the PSNR. However, when the kernel size increases, the upsampled images perform worse than the reconstructed images with regard to the PSNR, RMSE, and ERGAS. The SAM, on the other hand, has a slightly better performance in the upsampled images. Overall, the reconstructed images have a higher spatial quality than the upsampled images, as shown in Figure 2, where the largest improvement with regard to the upsampled images is clearly observed for a kernel size of 35×35.

### 3.2. Denoising

**Figure 3:**
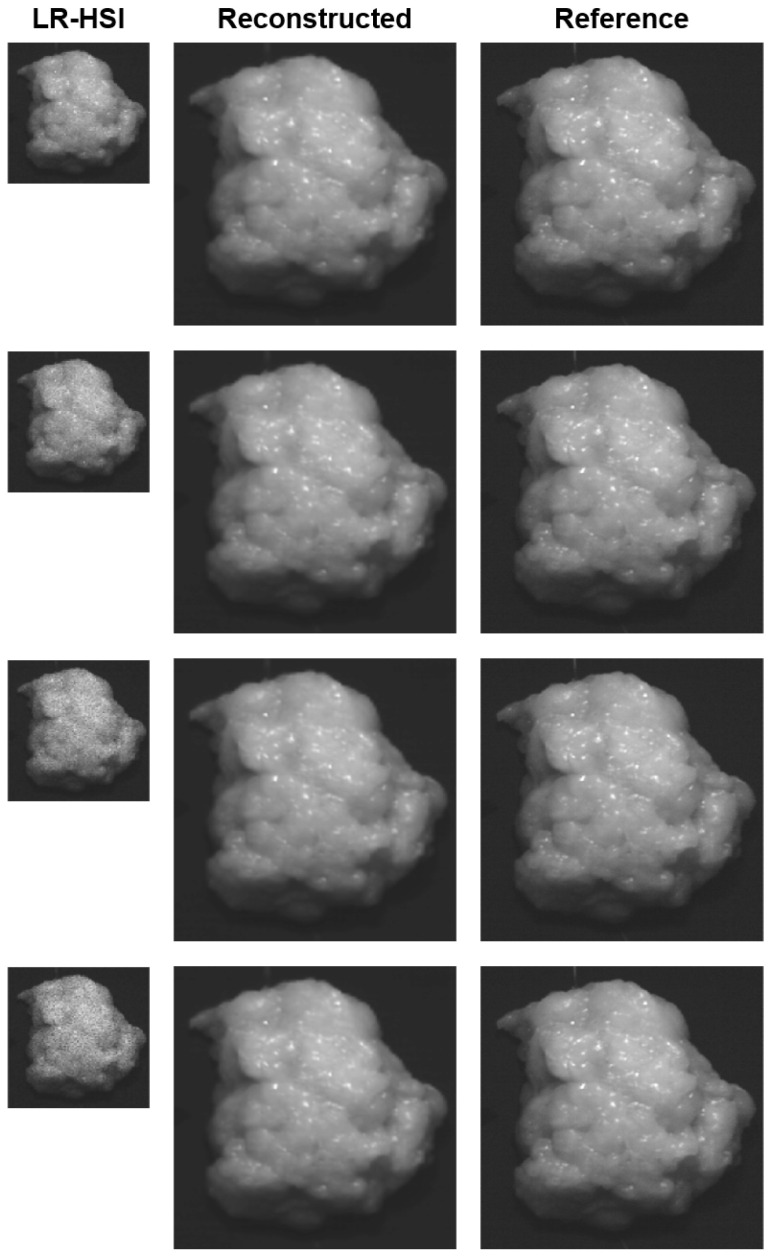
Noise experiment: example of a lumpectomy specimen after surgery. Gray color images are derived from the hyperspectral images with a wavelength band at 980 nm. From left to right: Noise LR-HSI, reconstructed HR-HSI, and reference HR-HSI. Noise was introduced into the spectra of the LR-HSI images to simulate the reduced spectral sensitivity of the camera sensor within the 980–1000 nm range. From top to bottom: Noise with a variance of 0.01, 0.03, 0.05, and 0.07.

The results of the noise experiment for one lumpectomy specimen are presented in Figure 3, in which a comparison is made between noise LR-HSI, reconstructed HR-HSI, and their reference HR-HSI images. For the noise images, the amount of noise ranges in a variance from 0.01 to 0.07. From this figure, it is evident that higher noise levels also lead to more noisy images. Consequently, this also affects the quality of the reconstruction, as indicated in Table 3, which presents the average performance metrics of the reconstruction with respect to the reference HR-HSI. Nevertheless, the results indicate that the differences among metrics remain relatively small when increasing the amount of noise in the image. Comparing the LR-HSI and reconstruction in Figure 3, it can be observed that the amount of noise has been entirely eliminated in all the reconstructed images, indicating an effective reconstruction process.

### 3.3. Dead Pixels Removal

**Figure 4:**
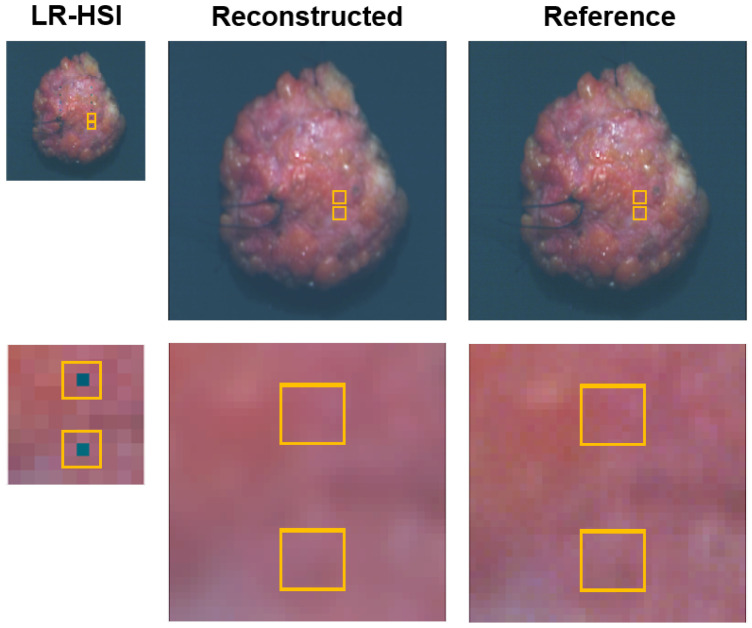
Dead pixels experiment. Pseudocolor images (wavelength bands at 650, 532, 473 nm) are derived from the hyperspectral images. Top row: lumpectomy specimen containing 12 dead pixels (black) on its resection surface, and associated image after reconstruction. Bottom row: enlarged view (magnification∼10×) of two delineated dead pixels highlighted in yellow.

**Figure 5:**
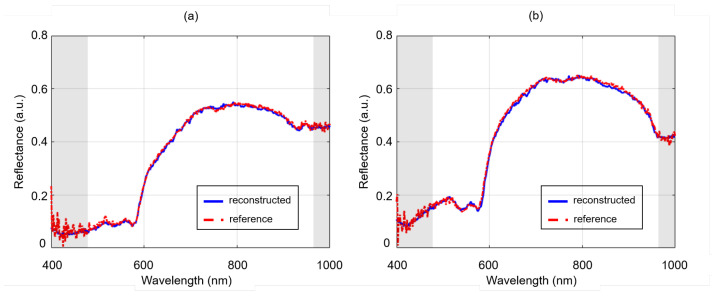
Comparison of reflectance spectra from two dead pixels that were restored in the reconstructed hyperspectral image. The upper pixel in the magnified image of Figure 4 corresponds to (**a**) and the lower pixel to (**b**). The shaded areas in gray represent the wavelengths at which the camera sensor exhibits reduced spectral sensitivity.

Figure 4 depicts the results of the dead pixels experiment on the resection surface of a lumpectomy specimen. On the first row, the most left HSI image represents the LR-HSI of the lumpectomy specimen with the synthetically created dead pixels followed by its corresponding reconstructed HR-HSI and reference HR-HSI image. For better visualization, on the second row, an enlarged view of the images is presented, revealing an area of two dead pixels delineated in yellow. From these images and the associated reflectance spectra depicted in Figure 5a (upper pixel) and Figure 5b (lower pixel), it is evident that the dead pixels can be effectively restored in the reconstructed HSI image. Considering these two dead pixels, the spectral shape of the associated reconstructed spectra are almost congruent to the reference spectra. Besides this, it can be noticed that the reference spectra exhibit higher noise levels within the wavelength range ∼400–450 nm and ∼950–1000 nm (delineated in gray) due to the lower spectral sensitivity of the camera sensor. In the reconstructed spectra, this noise has been entirely eliminated.

### 3.4. Specular Reflection Correction

**Figure 6:**
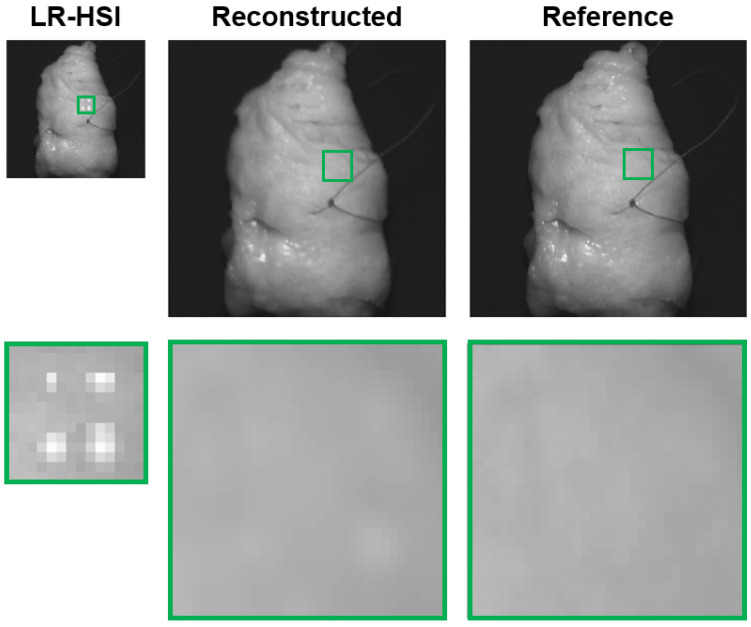
Specular reflection experiment. Gray color images derived from the hyperspectral images with a wavelength band at 705 nm. Top row: LR-HSI of lumpectomy specimen containing four regions of specular reflection in white, and associated result after reconstruction. Bottom row: enlarged view (magnification∼10×) of four regions with specular reflection.

**Figure 7:**
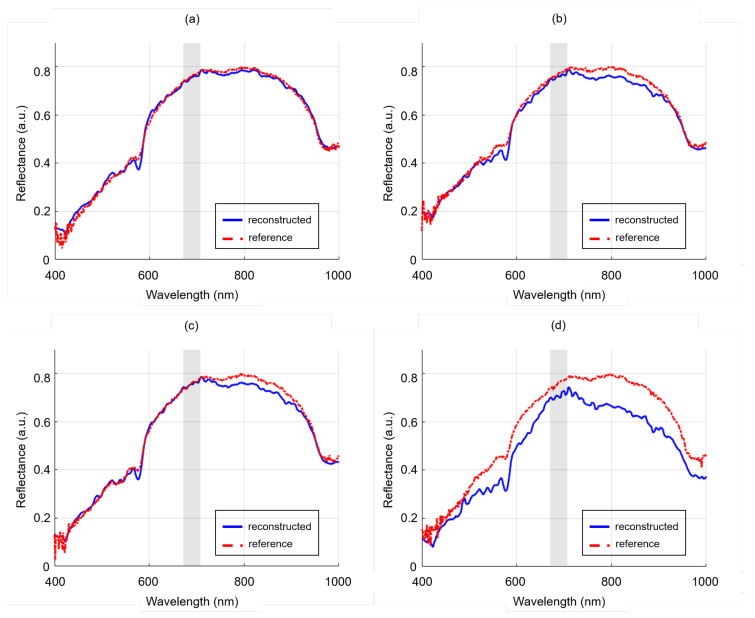
Comparison of reflectance spectra from four specular reflection regions that were restored in the reconstructed hyperspectral images. A comparison is made between the center pixels of the top-left (**a**), top-right (**b**), bottom-left (**c**), and bottom-right region (**d**) with a size of, respectively, 4, 9, 16, and 25 pixels. The regions are depicted in the magnified image of Figure 6. In each spectrum, the gray shaded area highlights the wavelength range associated with the specular reflection (675–705 nm).

The outcome of the specular reflection experiment conducted on a lumpectomy sample is illustrated in Figure 6. The first image depicts four square regions of created specular reflection in white, containing a total of, respectively, 4, 9, 16, and 25 pixels. A closer view of the four regions is shown on the second row (delineated in green). In the reconstructed image, it becomes apparent that the smaller regions with specular reflection are almost entirely restored spatially. However, the larger regions with specular reflection still exhibit some visibility, although very subtle. In Figure 7, the reflectance spectra of these four specular reflection regions are presented. Specifically, Figure 7a,b show the center pixel of, respectively, the top-left and top-right region, whereas Figure 7c,d depict the center of, respectively, the bottom-left and bottom-right region. In all spectra, the shaded gray region delineates the wavelength interval corresponding to the specular reflection (675–705 nm). The reconstructed spectrum from the smallest region (four pixels), nearly resembles the reference spectrum, confirming the network’s ability to restore the lost information as a result of the specular reflection. For the larger regions with specular reflection, the reconstructed spectra display greater deviations from the reference spectra. Nevertheless, these deviations remain relatively small for the middle-size specular reflection regions (9 and 16 pixels), in line with the spatial findings presented in Figure 6. However, for the largest specular reflection region (25 pixels), the disparity between the reconstructed and reference spectra is more pronounced in both spectral shape and intensity.

## 4. Discussion

To improve the outcome of breast-conserving surgeries, it is essential to have an evaluation technique that can provide immediate feedback to the surgeon regarding the margins of the resected tissue. Hyperspectral imaging emerges as a promising margin assessment technique during surgery. However, to be implicated intraoperatively, it should be fast and capable of yielding high-quality images. Therefore, in this study, we spatially and spectrally reconstructed the hyperspectral images of breast lumpectomy specimens. By employing this deep learning reconstruction framework, we demonstrated the ability to successfully produce high-resolution HSI images from low-resolution HSI images combined with high-resolution RGB images, which translates to a lower data acquisition time during surgery while maintaining a high image quality. Furthermore, we showed the capability to overcome complex issues such as blur, noise, dead pixels, and specular reflections. As these issues commonly arise during the analysis of hyperspectral images, solving them is essential to facilitate an accurate tissue classification during surgery.

While training the model, we implemented early stopping based on the PSNR and SAM metrics calculated on the validation set for optimization purposes. We applied an early stopping strategy with both metrics to reduce the impact of random variations and enhance the overall evaluation process before making model adjustments. Occasionally, a cropped image might include a significant fraction of the black background, which could lead to a higher PSNR in comparison to the tissue region. Relying solely on the PSNR metric could consequently lead to a distorted representation of the actual performance. By incorporating an early stopping that considers the PSNR as well as the SAM, both metrics must be satisfied, thus ensuring a more robust method for model refinement, diminishing the influence of incidental variations and less prone to overfitting.

We achieved the highest reconstruction performance on the Gaussian blur images with a kernel-size of 5 × 5. This aligns with our expectations, since the model was trained using images of identical kernel size. Furthermore, the images only exhibited a slight blur with respect to their reference image, which may explain the subtle difference in performance between the reconstructed and upsampled images (Table 2). When there is a high degree of similarity among the pixels within an image, the process of upsampling the low-resolution HSI image only becomes more easy. In such cases, missing pixels can be effectively replaced through bilinear interpolation, which relies on calculating the weighted average of neighboring pixels exclusively. Hence, this might explain the slightly improved spectral reconstruction score observed in the upsampled images compared to the reconstructed images.

To improve the spectral reconstruction in the proposed method, we could consider adjusting the early stopping strategy by assigning higher weights to the SAM metric than the PSNR metric. This would ensure that the model would be updated only when higher SAM values are achieved. However, it is important to notice that this adjustment might have an impact on the spatial quality of the images. Therefore, it is crucial to make a careful choice regarding the relative significance of these two factors. For images with a more substantial blurring (Figure 2), we also showed that the reconstruction outperforms the upsampling approach. This is because the trained reconstruction network can take advantage of both the spatial and spectral information that are present in the preliminary fused RGB-HSI, resulting in the most optimal reconstruction quality. In contrast, the upsampling approach fully relies on calculating the weighted average of the nearest pixels in the LR-HSI, which are already affected by the blur. Thus, when the level of distortion in the images increases, e.g., because of motion artifacts during surgery, it is not surprising that the reconstruction will perform substantially better than the upsampling approach as the ability to resize the images adequately using bilinear interpolation decreases.

The results in Figure 3 clearly illustrate that higher levels of noise correspond to more visually noisy images. This observation aligns with the expected behavior, as increased noise levels generally decrease image quality. Comparing the LR-HSI images with their reconstructed images, it is evident that the reconstruction process has effectively eliminated the noise in all the reconstructed images. This is also confirmed by the outcomes presented in Table 3, where all metrics display a consistently high performance when comparing the reconstructed images with their reference HR-HSI. Furthermore, spectrally, a distinct improvement can be observed at the extremities of the wavelength range where the noise was (synthetically) introduced (Figure 5). Given the unavoidable presence of noise in these wavelength regions due to the reduced spectral sensitivity of the camera sensor, this denoising result effectively demonstrates the reconstruction network’s potential to improve both the image quality and associated spectra. In particular, in the context of margin assessment in which both types of information are crucial [12], this could lead to a more reliable and accurate analysis.

The occurrence of dead pixels accounts for approximately 1% in HSI sensors [35]. Although this percentage does not appear substantially, single defective pixels lead to the loss of valuable information at specific wavelengths. This could be critical, especially when it pertains to wavelengths essential for tissue classification. On top of that, dead pixels also tend to rise in number over time. Figure 4 demonstrates that the reconstruction network performs well in restoring dead pixels. The corresponding reconstructed spectra (Figure 5) closely resemble those of the reference, indicating an effective reconstruction. This is reasonable as it involves the reconstruction of individual pixels.

When considering more extensive regions of lost information in the spectral domain, such as areas exhibiting specular reflection, as shown in Figure 6, the reconstruction network also demonstrates its effectiveness. It should be emphasized that a proper reconstruction can only occur when the HR-RGB is free from specular reflection, but this condition can be easily met by attaching a polarization filter to the lens of the regular color camera. Using a polarization filter for a regular color camera rather than a hyperspectral camera, is more cost-effective and will have no impact on the reflectance spectra. When considering the spectra of the regions, (Figure 7) we showed that smaller regions with specular reflection can be reconstructed better than larger regions. Typically, the regions exhibiting specular reflection are surrounded by pixels that either show no saturation or less saturation in comparison to pixels located in the center; as a result, these surrounding pixels preserve a greater amount of information. When dealing with a larger specular reflection region containing more saturated pixels, a substantial portion of the information is lost, making it more challenging to achieve accurate pixel reconstruction. This observation suggests that there exists a limitation on the size of specular reflections that the reconstruction network can effectively reconstruct. However, it is worth noting that the model was not trained on specular reflection, thus still leaving room for improvement. Nonetheless, since areas with specular reflection frequently occur on tissue surfaces, not only in lumpectomy specimens, but also in specimens with smoother surfaces such as the colon, rectum, or brain, employing the network to reconstruct these otherwise lost regions would already represent a significant improvement in detecting potential suspicious tumor tissue more rapidly.

Given the PSNR values of the reconstructed Gaussian blur images, our results are in line with those reported in the literature [33,41]. As indicated by Chervyakov et al. [42], our results can be regarded as high-quality medical images. Zhang et al. achieved PSNR values ranging from 35.26 to 43.49. Although comparable, it should be noticed that the authors trained, validated, and tested on the same images, which in our case, would lead to a bias in the results. Furthermore, their dataset consisted of satellite images without any unnecessary background. So with each epoch, only useful information was included during training, implying that training on tissue only could potentially increase the performance.

Li et al. [41] achieved PSNR values between 27.20 and 49.70 on different medical datasets. In their study, they used publicly available datasets with HSI images from the brain and oral/dental region. On the brain dataset they showed a similar performance as our study with PSNR values between 37.40 and 38.30. However, when using the dental dataset, they demonstrated a notably higher performance with PSNR values within the range of 47.10–49.70. These differences in performance might be explained by the various tissue types that were present in these datasets. In case of the dental dataset, the improved reconstruction results might be due to a greater contrast between the teeth and the surrounding oral region, along with reduced noise levels. Additionally, the authors conducted an experiment in which they trained their model on the dental dataset and tested it on the brain dataset. This cross-dataset evaluation resulted in a performance drop, with PSNR values ranging from 27.20 to 33.60. This decrease in performance is logically explained by the dissimilarities between the datasets, including differences in hardware (various hyperspectral cameras) and tissue types.

In another study conducted by Tang et al. [43], the impact of down- and upsampling on the performance metrics was demonstrated. The authors varied the downsampling factor between 8 and 32 resulting in a RMSE and SAM between, respectively, 1.06–2.18 and 1.64–2.07 on the Pavia Center dataset. Avagyan et al. [32] showed a decrease in PSNR from 37.49 to 35.39 on the same dataset when increasing the downsampling ratio from 4 to 32. The slight decline in performance metrics implies the potential of further downsampling the HSI data so that, in practice, images can be used with even a lower resolution and consequently shorter acquisition time. However, increasing the downsampling factor too much will increase the risk of missing important regions, such as small tumors, which may be filtered out as a result of this high factor. As we now used RGB images taken from a line-scanning dataset, the spatial resolution is still relatively low compared to that of a regular color camera. Nevertheless, for future studies with an actual color camera, we could use the reconstruction network to take advantage of the high-resolution RGB image, and theoretically reconstruct a hyperspectral image with a spatial resolution 32 times higher than the current settings. Yet, it is essential to consider that an increased resolution image will also affect the time required to perform a tissue classification.

The reconstructed Gaussian blur images exhibited a slight decrease in performance relative to the increase in blurriness. Even though the blurred images could be reconstructed well, the reconstruction network was solely trained on lumpectomy images with a Gaussian kernel size of 5 × 5 and standard-deviation of 10. Therefore, the reconstruction abilities might improve when the network is trained on images with a more substantial blurring. This also holds for the images with noise, dead pixels, and specular reflection, on which the model was not trained at all. When incorporating this variety of images in the training set, this might potentially increase the performance of the reconstruction network.

Without reconstruction, a visual line-scanning camera requires approximately 40 s to acquire an adequate image of a single resection side of the lumpectomy specimen. This means that if the entire resection surface was captured and analyzed, the process would require no more than 5–10 min in total. For tissue classification of ex vivo resection specimens only, this time frame is relatively fast, particularly when compared to other margin assessment techniques with a comparable diagnostic performance [12], such as frozen section analysis. Frozen section analysis typically takes about 20–30 min and only examines a small biopsy sample from the entire resection surface [44]. However, for in vivo tissue classification, the acquisition time of a line-scanning camera would strongly increase the risk of motion artifacts. For intraoperative purposes, a snapshot camera would therefore be more convenient as it offers faster, (near) real-time data acquisition, and a compact size for close proximity to the patient. Currently, with the latest advancements in commercial snapshot cameras, speeds of more than 20 frames per second can be achieved, indicating the possibility of real-time feedback during surgery. Nevertheless, it is important to note that the spatial and spectral resolutions of these snapshot cameras still lag behind to those of line-scanning hyperspectral cameras [23]. Depending on the specifications of the currently available snapshot cameras, the spatial resolution is approximately 3–4 times lower, and the spectral resolution is also around 2–3 times lower compared to line-scanning cameras. As a higher spectral resolution comes at the cost of spatial resolution, these types of cameras have a lower spatial resolution, which could hamper the visualization and interpretation of surgeons during BCS. In a surgical setting, having clear, high-resolution images is crucial for providing accurate guidance and enabling precise decision-making. When the HSI images lack the necessary detail and clarity, this could lead to reduced precision in tumor detection and longer procedure times. Furthermore, when motion artifacts occur, the impact would be even larger, as hyperspectral cameras are designed to be stationary. By synchronizing the low-resolution snapshot camera with a regular high-resolution color camera equipped with autofocus capabilities, and utilizing our proposed reconstruction framework, the low-resolution images of the snapshot camera can be easily reconstructed to high-resolution images within approximately 5 s on a computer workstation. With more recent advancements in GPU cards and GPU computing, the process may be accelerated further, potentially achieving real-time reconstruction. By incorporating the analysis of the lumpectomy specimen into this workflow, the time required will be less than 30 s per resection side. This enables a quick and adequate margin assessment during cancer surgery.

In this study, we have demonstrated the effectiveness of employing our reconstruction framework for breast-conserving surgery. This approach, when integrated with a snapshot hyperspectral camera and a regular color camera in the surgical workflow, not only optimizes the imaging process but also ensures that surgeons have access to high-resolution information, which promotes more precise decision-making during the surgery. Although this work represented a feasibility study, primarily to bring HSI one step closer to clinical implementation, it holds promise for future research. The future research should focus on evaluating the performance of the reconstruction network in a real surgical setting, where both an actual snapshot camera and a standard color camera are synchronized to assess lumpectomy specimen resection margins in vivo. Considering the margin assessment, it is essential for subsequent analysis to compare the ability to detect tumors within the resection margins both before and after image reconstruction. Furthermore, we would recommend training the model on a lumpectomy dataset with snapshot images instead of line-scanning images, as snapshot images typically exhibit a reduced number of wavelength bands. Given the challenges associated with obtaining such a dataset, it would be interesting to explore the extent to which the reconstruction network can maintain a high level of performance on the current dataset, even when a substantial number of bands is removed.

## 5. Conclusions

In this study, we investigated the feasibility of using a spatial–spectral reconstruction framework to yield high-resolution hyperspectral images of breast lumpectomy specimens with the purpose of enabling accurate tissue discrimination during breast-conserving surgery. Using this approach, low-resolution HSI images and high-resolution RGB images could be successfully reconstructed into high-resolution HSI images. Additionally, we showed the network’s capability to solve complex issues, often encountered in the analysis of hyperspectral images, such as noise, dead pixels, specular reflections, and blur due to motion artifacts. With the results of this study, we demonstrated the ability to perform fast data acquisition during surgery while maintaining a high image quality, thereby providing the opportunity to ultimately facilitate an adequate margin assessment with hyperspectral imaging. Although the images of a line-scanning camera were used to synthetically generate low-resolution HSI images from a snapshot hyperspectral camera and high-resolution RGB images from a regular color camera, the obtained results hold promise for future research to assess the network’s performance in a real surgical scenario where an actual snapshot camera and simple color camera are synchronized to assess the resection margins of lumpectomy specimens in vivo.

## Figures and Tables

**Table 2:** Performance metrics blurring experiment: Comparison reconstructed HR-HSI and upsampled LR-HSI with reference HR-HSI images (mean ± std).

	Metrics	Kernel 5 × 5	Kernel 15 × 15	Kernel 25 × 25	Kernel 35 × 35
Reconstructed	PSNR	38.6 ± 2.13	37.4 ± 2.19	36.4 ± 2.27	35.8 ± 2.32
	RMSE	0.012 ± 0.004	0.013 ± 0.004	0.014 ± 0.004	0.015 ± 0.005
	ERGAS	0.100 ± 0.015	0.105 ± 0.017	0.115 ± 0.020	0.123 ± 0.023
	SAM	1.34 ± 0.136	1.43 ± 0.166	1.54 ± 0.194	1.61 ± 0.212
Upsampled	PSNR	37.5 ± 1.93	33.7 ± 2.05	32.2 ± 2.16	31.5 ± 2.24
	RMSE	0.011 ± 0.003	0.016 ± 0.005	0.019 ± 0.006	0.021 ± 0.007
	ERGAS	0.091 ± 0.015	0.131 ± 0.024	0.157 ± 0.032	0.172 ± 0.036
	SAM	1.13 ± 0.125	1.36 ± 0.176	1.51 ± 0.213	1.60 ± 0.232

**Table 3:** Performance metrics noise experiment: Comparison reconstructed HR-HSI with reference HR-HSI images per noise variance $\sigma^{2}$ (mean ± std).

Metrics	0.01 $\sigma^{2}$	0.03 $\sigma^{2}$	0.05 $\sigma^{2}$	0.07 $\sigma^{2}$
PSNR	38.5 ± 2.14	37.7 ± 3.59	37.2 ± 4.50	35.4 ± 6.61
RMSE	0.012 ± 0.003	0.014 ± 0.008	0.016 ± 0.011	0.022 ± 0.021
ERGAS	0.096 ± 0.016	0.108 ± 0.042	0.121 ± 0.063	0.168 ± 0.130
SAM	1.34 ± 0.133	1.34 ± 0.133	1.35 ± 0.132	1.35 ± 0.132

## Data Availability

The data presented in this study are available on request from the corresponding author. The data are not publicly available.

## References

[1] Langhans L., Jensen M.B., Talman M.L.M., Vejborg I., Kroman N., Tvedskov T.F. (2017). Reoperation rates in ductal carcinoma in situ vs. invasive breast cancer after wire-guided breast-conserving surgery. JAMA Surg..

[2] Merrill A.L., Coopey S.B., Tang R., McEvoy M.P., Specht M.C., Hughes K.S., Gadd M.A., Smith B.L. (2016). Implications of new lumpectomy margin guidelines for breast-conserving surgery: Changes in reexcision rates and predicted rates of residual tumor. Ann. Surg. Oncol..

[3] Merrill A.L., Tang R., Plichta J.K., Rai U., Coopey S.B., McEvoy M.P., Hughes K.S., Specht M.C., Gadd M.A., Smith B.L. (2016). Should new “no ink on tumor” lumpectomy margin guidelines be applied to ductal carcinoma in situ (DCIS)? A retrospective review using shaved cavity margins. Ann. Surg. Oncol..

[4] Alrahbi S., Chan P.M., Ho B.C., Seah M.D., Chen J.J., Tan E.Y. (2015). Extent of margin involvement, lymphovascular invasion, and extensive intraductal component predict for residual disease after wide local excision for breast cancer. Clin. Breast Cancer.

[5] Landercasper J., Ellis R.L., Mathiason M.A., Marcou K.A., Jago G.S., Dietrich L.L., Johnson J.M., De Maiffe B.M. (2010). A community breast center report card determined by participation in the national quality measures for breast centers program. Breast J..

[6] Van Den Bruele A.B., Jasra B., Smotherman C., Crandall M., Samiian L. (2018). Cost-effectiveness of surgeon performed intraoperative specimen ink in breast conservation surgery. J. Surg. Res..

[7] Taghian A., Mohiuddin M., Jagsi R., Goldberg S., Ceilley E., Powell S. (2005). Current perceptions regarding surgical margin status after breast-conserving therapy: Results of a survey. Ann. Surg..

[8] Smitt M.C., Nowels K., Carlson R.W., Jeffrey S.S. (2003). Predictors of reexcision findings and recurrence after breast conservation. Int. J. Radiat. Oncol. Biol. Phys..

[9] Qiu S.Q., Dorrius M.D., de Jongh S.J., Jansen L., de Vries J., Schröder C.P., Zhang G.J., de Vries E.G., van der Vegt B., van Dam G.M. (2018). Micro-computed tomography (micro-CT) for intraoperative surgical margin assessment of breast cancer: A feasibility study in breast conserving surgery. Eur. J. Surg. Oncol..

[10] Mojahed D., Ha R.S., Chang P., Gan Y., Yao X., Angelini B., Hibshoosh H., Taback B., Hendon C.P. (2020). Fully automated postlumpectomy breast margin assessment utilizing convolutional neural network based optical coherence tomography image classification method. Acad. Radiol..

[11] Li R., Wang P., Lan L., Lloyd F.P., Goergen C.J., Chen S., Cheng J.X. (2015). Assessing breast tumor margin by multispectral photoacoustic tomography. Biomed. Opt. Express.

[12] Jong L.J.S., de Kruif N., Geldof F., Veluponnar D., Sanders J., Peeters M.J.T.V., van Duijnhoven F., Sterenborg H.J., Dashtbozorg B., Ruers T.J. (2022). Discriminating healthy from tumor tissue in breast lumpectomy specimens using deep learning-based hyperspectral imaging. Biomed. Opt. Express.

[13] Jong L.J.S., Post A.L., Veluponnar D., Geldof F., Sterenborg H.J., Ruers T.J., Dashtbozorg B. (2023). Tissue Classification of Breast Cancer by Hyperspectral Unmixing. Cancers.

[14] Jong L.J., de Kruif N., Geldof F., Veluponnar D., Sanders J., Peeters M.J.V., van Duijnhoven F., Sterenborg H., Dashtbozorg B., Ruers T. Resection margin assessment in breast lumpectomy specimens using deep learning-based hyperspectral imaging (Conference Presentation). Proceedings of the Advanced Biomedical and Clinical Diagnostic and Surgical Guidance Systems XXI, SPIE.

[15] Kho E., Dashtbozorg B., De Boer L.L., Van de Vijver K.K., Sterenborg H.J., Ruers T.J. (2019). Broadband hyperspectral imaging for breast tumor detection using spectral and spatial information. Biomed. Opt. Express.

[16] Kho E., Dashtbozorg B., Sanders J., Vrancken Peeters M.J.T., van Duijnhoven F., Sterenborg H.J., Ruers T.J. (2021). Feasibility of ex vivo margin assessment with hyperspectral imaging during breast-conserving surgery: From imaging tissue slices to imaging lumpectomy specimen. Appl. Sci..

[17] Keating J.J., Fisher C., Batiste R., Singhal S. (2016). Advances in intraoperative margin assessment for breast cancer. Curr. Surg. Rep..

[18] Barberio M., Collins T., Bencteux V., Nkusi R., Felli E., Viola M.G., Marescaux J., Hostettler A., Diana M. (2021). Deep learning analysis of in vivo hyperspectral images for automated intraoperative nerve detection. Diagnostics.

[19] Felli E., Al-Taher M., Collins T., Nkusi R., Felli E., Baiocchini A., Lindner V., Vincent C., Barberio M., Geny B. (2021). Automatic liver viability scoring with deep learning and hyperspectral imaging. Diagnostics.

[20] Wang P., Li P., Yin M., Li Y., Wu J. (2020). Burn wound assessment system using near-infrared hyperspectral imaging and deep transfer features. Infrared Phys. Technol..

[21] Eggert D., Bengs M., Westermann S., Gessert N., Gerstner A.O., Mueller N.A., Bewarder J., Schlaefer A., Betz C., Laffers W. (2022). In vivo detection of head and neck tumors by hyperspectral imaging combined with deep learning methods. J. Biophotonics.

[22] Fabelo H., Halicek M., Ortega S., Shahedi M., Szolna A., Piñeiro J.F., Sosa C., O’Shanahan A.J., Bisshopp S., Espino C. (2019). Deep learning-based framework for in vivo identification of glioblastoma tumor using hyperspectral images of human brain. Sensors.

[23] Halicek M., Fabelo H., Ortega S., Callico G.M., Fei B. (2019). In-vivo and ex vivo tissue analysis through hyperspectral imaging techniques: Revealing the invisible features of cancer. Cancers.

[24] Blanch-Perez-del Notario C., Luthman S., Lefrant R., Gonzalez P., Lambrechts A. Compact high-speed snapshot hyperspectral imager in the SWIR range (1.1–1.65 nm) and its potential in sorting/recycling industry. Proceedings of the Algorithms, Technologies, and Applications for Multispectral and Hyperspectral Imaging XXVIII, SPIE.

[25] Buttingsrud B., Alsberg B.K. (2006). Superresolution of hyperspectral images. Chemom. Intell. Lab. Syst..

[26] Wang L., Zhao C. (2016). Hyperspectral Image Processing.

[27] Akhtar N., Shafait F., Mian A. Bayesian sparse representation for hyperspectral image super resolution. Proceedings of the IEEE Conference on Computer Vision and Pattern Recognition.

[28] Kawakami R., Matsushita Y., Wright J., Ben-Ezra M., Tai Y.W., Ikeuchi K. High-resolution hyperspectral imaging via matrix factorization. Proceedings of the CVPR 2011, IEEE.

[29] Xie W., Jia X., Li Y., Lei J. (2019). Hyperspectral image super-resolution using deep feature matrix factorization. IEEE Trans. Geosci. Remote Sens..

[30] Ma L., Rathgeb A., Mubarak H., Tran M., Fei B. (2022). Unsupervised super-resolution reconstruction of hyperspectral histology images for whole-slide imaging. J. Biomed. Opt..

[31] Li Y., Hu J., Zhao X., Xie W., Li J. (2017). Hyperspectral image super-resolution using deep convolutional neural network. Neurocomputing.

[32] Avagyan S., Katkovnik V., Egiazarian K. (2022). Modified SSR-NET: A Shallow Convolutional Neural Network for Efficient Hyperspectral Image Super-Resolution. Front. Remote Sens..

[33] Zhang X., Huang W., Wang Q., Li X. (2020). SSR-NET: Spatial–spectral reconstruction network for hyperspectral and multispectral image fusion. IEEE Trans. Geosci. Remote Sens..

[34] Maity A., Pattanaik A., Sagnika S., Pani S. A comparative study on approaches to speckle noise reduction in images. Proceedings of the 2015 International Conference on Computational Intelligence and Networks, IEEE.

[35] Dorrepaal R., Malegori C., Gowen A. (2016). Tutorial: Time series hyperspectral image analysis. J. Infrared Spectrosc..

[36] Helmy A., El-Tawel G.S. (2015). An integrated scheme to improve pan-sharpening visual quality of satellite images. Egypt. Inform. J..

[37] Chai T., Draxler R.R. (2014). Root mean square error (RMSE) or mean absolute error (MAE). Geosci. Model Dev. Discuss..

[38] Poobathy D., Chezian R.M. (2014). Edge detection operators: Peak signal to noise ratio based comparison. Int. J. Image Graph. Signal Process..

[39] Renza D., Martinez E., Arquero A. (2012). A new approach to change detection in multispectral images by means of ERGAS index. IEEE Geosci. Remote Sens. Lett..

[40] Kuching S. (2007). The performance of maximum likelihood, spectral angle mapper, neural network and decision tree classifiers in hyperspectral image analysis. J. Comput. Sci..

[41] Li P., Ebner M., Noonan P., Horgan C., Bahl A., Ourselin S., Shapey J., Vercauteren T. (2022). Deep learning approach for hyperspectral image demosaicking, spectral correction and high-resolution RGB reconstruction. Comput. Methods Biomech. Biomed. Eng. Imaging Vis..

[42] Chervyakov N., Lyakhov P., Nagornov N. (2020). Analysis of the quantization noise in discrete wavelet transform filters for 3D medical imaging. Appl. Sci..

[43] Tang S., Xu Y., Huang L., Sun L. (2019). Hyperspectral Image Super-Resolution via Adaptive Dictionary Learning and Double L 1 Constraint. Remote Sens..

[44] Esbona K., Li Z., Wilke L.G. (2012). Intraoperative imprint cytology and frozen section pathology for margin assessment in breast conservation surgery: A systematic review. Ann. Surg. Oncol..

